# When and how ruling out cystic fibrosis in adult patients with bronchiectasis

**DOI:** 10.1186/s40248-018-0142-7

**Published:** 2018-08-09

**Authors:** Andrea Gramegna, Stefano Aliberti, Manuela Seia, Luigi Porcaro, Vera Bianchi, Carlo Castellani, Paola Melotti, Claudio Sorio, Enza Consalvo, Elisa Franceschi, Francesco Amati, Martina Contarini, Michele Gaffuri, Luca Roncoroni, Barbara Vigone, Angela Bellofiore, Cesare Del Monaco, Martina Oriano, Leonardo Terranova, Maria Francesca Patria, Paola Marchisio, Baroukh M. Assael, Francesco Blasi

**Affiliations:** 1 Department of Pathophysiology and Transplantation, University of Milan, Internal Medicine Department, Respiratory Unit and Cystic Fibrosis Adult Center, Fondazione IRCCS Ca’ Granda Ospedale Maggiore Policlinico, Via Francesco Sforza 35, 20122 Milan, Italy; 2 Medical Genetics Laboratory, Fondazione IRCCS Ca’ Granda Ospedale Maggiore Policlinico, 20122 Milan, Italy; 3 UOSD Genetica Medica, Medical Genetics Unit, Fondazione IRCCS Ca’ Granda Ospedale Maggiore Policlinico, 20122 Milan, Italy; 4 Centro Fibrosi Cistica, Azienda Ospedaliera Universitaria Integrata, Verona, Italy; 5 Dipartimento di Patologia e Diagnostica, Università di Verona, Verona, Italy; 6 Department of Otolaryngology and Head and Neck Surgery, Fondazione IRCCS Ca’ Granda Ospedale Maggiore Policlinico, Department of Clinical Sciences and Community Health, University of Milan, 20122 Milan, Italy; 7 Scleroderma Unit, Referral Center for Systemic Autoimmune Diseases, Fondazione IRCCS Ca’ Granda Ospedale Maggiore Policlinico di Milano, 20122 Milan, Italy; 8 Pediatric Highly Intensive Care Unit, Department of Pathophysiology and Transplantation, Università degli Studi di Milano, Fondazione IRCCS Ca’ Granda Ospedale Maggiore Policlinico, 20122 Milan, Italy; 9 Molecular Medicine Department, University of Pavia, Viale Taramelli 3/b, 27100 Pavia, Italy

**Keywords:** Bronchiectasis, CFTR, Etiological screening, CFTR gene analysis, Sweat test

## Abstract

**Background:**

Bronchiectasis is the final result of different processes and most of the guidelines advocate for a careful evaluation of those etiologies which might be treated or might change patients’ management, including cystic fibrosis (CF).

**Main body:**

CFTR mutations have been reported with higher frequency in bronchiectasis population. Although ruling out CF is considered as a main step for etiological screening in bronchiectasis, CF testing lacks of a standardized approach both from a research and clinical point of view. In this review a list of most widely used tests in CF is provided.

**Conclusions:**

Exclusion of CF is imperative for patients with bronchiectasis and CFTR testing should be implemented in usual screening for investigating bronchiectasis etiology. Physicians taking care of bronchiectasis patients should be aware of CFTR testing and its limitations in the adult population. Further studies on CFTR expression in human lung and translational research might elucidate the possible role of CFTR in the pathogenesis of bronchiectasis.

## Background

Bronchiectasis is a chronic respiratory disease characterized by a permanent dilation of the bronchi associated with cough, daily sputum production and recurrent episodes of respiratory infections [1]. Although studies probably underestimate the true prevalence of bronchiectasis, recent literature suggests bronchiectasis as far from being a rare disease [2]. Different European datasets describes a prevalence of bronchiectasis ranging from 67 to 566 *per* 100,000 with a significant increase with age and in females [3–5]. These records promoted a renewed awareness of this condition as demonstrated by an increase in research activity and the development of international registries over the past few years [6–8].

The pathophysiology of bronchiectasis might start with either structural airway damage or impaired mucociliary clearance, leading to subsequent chronic bacterial infection and a neutrophilic inflammation, which perpetuates this vicious cycle [9]. Bronchiectasis is the final result of different processes, which explains the extreme heterogeneity of the disease [8]. The clinical spectrum of bronchiectasis includes radiological abnormalities in almost asymptomatic patients as well as diffuse respiratory involvement with severe functional and radiological impairment in frequent exacerbations with high morbidity and mortality. In view of the extreme variety of this condition, different methods have been proposed in literature to cluster discrete groups of patients sharing common clinical features, biological substrates and outcomes [10–12]. Conflicting results have been proposed with the above analysis and most of the scientific community argues on the implementation of this research in clinical practice [13].

Besides clinical phenotypes, one of the imperative steps in the management of patients with bronchiectasis still remains the evaluation of the underlying etiologies [14]. Most of the international guidelines advocate for a careful evaluation of possible etiologies of bronchiectasis especially those which might be treated or might change patients’ management [15, 16]. The recent guidelines published by the European Respiratory Society suggests a minimum bundle of etiological tests to be performed in adults with a new diagnosis of bronchiectasis, including differential blood count, serum immunoglobulins and testing for allergic bronchopulmonary aspergillosis [14]. This conditional recommendation is based on very low quality of evidence and practices to investigate bronchiectasis etiology are highly heterogeneous across European countries [17, 18]. Furthermore, a deep analysis of bronchiectasis etiology might be limited by an extensive and costly bundle of tests, which might be run only in tertiary care centers [17, 18]. Finally, experts agree neither on the definition of some etiologies of bronchiectasis (e.g. post-infective) nor the significance of “cause” vs. “association” vs. “overlap” between bronchiectasis and other diseases (e.g. COPD) [19].

## Why rule out cystic fibrosis in adults with bronchiectasis?

Although the scenario concerning the investigation of bronchiectasis etiology is still evolving, both clinical and scientific communities agree on the need to rule out cystic fibrosis (CF). CF is an autosomic recessive disease caused by at least two mutations of a gene encoding a transmembrane chloride channel called Cystic Fibrosis Transmembrane Conductance Regulator (CFTR) involved in regulation of liquid volume and anions on epithelial surfaces.

Ruling out CF in bronchiectasis patients is important in view of clinical, social-economical and psychological consequences. From a clinical perspective, CF is a systemic disease with extra-respiratory manifestations, which benefits from a specific management with a favorable impact on patients’ prognosis and quality of life [20]. CF patients have access to respiratory therapies that patients with bronchiectasis have not formal indication for and those with specific mutations might benefit from new CFTR modulator therapies. From a social-economical perspective, CF patients might benefit from a tertiary-care management in specialized centers, have access to free care in some countries or benefit from a special legislation and proper health insurance plans. From a psychological perspective, CF is a chronic, life-limiting disease with implications for patients’ life plans. CF patients have an increased risk of affected children and might benefit from a referral to genetic counseling.

Although the majority of patients with CF are identified by newborn screening or during childhood, late diagnosis of adults is not infrequent [21]. However, testing for CF is not included in the *minimum* bundle of investigations recommended by international guidelines on the management of bronchiectasis patients and the exclusion of CF is suggested only in the presence of specific clinical features [14]. These include early age of symptom onset, diffuse bronchiectasis with predominant upper lobe distribution on CT scan, early presence of *Staphylococcus aureus, Pseudomonas aeruginosa* or *Burkholderia cepacia* in respiratory cultures, upper airway disease like polyps or chronic sinusitis, extra-respiratory involvement such as recurrent pancreatitis and maldigestion, or male infertility, see Table 1 [14, 16, 22–24].

**Table 1:** When and how rule out CF according to existing bronchiectasis guidelines

Guidelines	When	How
ERS 2017 [14]	Selected adults including those with any of the following: young adults; upper lobe predominance of bronchiectasis on chest CT; the presence of nasal polyposis and/or chronic rhinosinusitis; recurrent pancreatitis; male primary infertility and/or malabsorption	Sweat chloride, other biomarkers of CFTR activity and CFTR mutation analysis
Pulmonology Portoguese Society, 2016 [16]	All children and selected adults (no further specified)	Two measurements of sweat chloride and CFTR mutation analysis for
British Thoracic Society, 2010 [22]	All children and selected adults including those with any of the following: adults up to the age of 40; age at presentation > 40 years and no other identified cause; persistent isolation of *Staphylococcus aureus* in the sputum; features of malabsorption; male primary infertility; upper lobe bronchiectasis; a history of childhood steatorrhoea.	Two measurements of sweat chloride and CFTR mutation analysis
Normativa SEPAR, 2008 [23]	Selected adults	Sweat chloride, nasal potential difference, CFTR mutation analysis test
Thoracic Society of Australia and New Zealand, 2015 [24]	All children and selected adults (no further specified)	Sweat test

In addition, there is no agreement across bronchiectasis guidelines on which diagnostic test should be used to exclude CF. While the majority of guidelines refers to sweat chloride measurement and *CFTR* genetic analysis as diagnostic standards, no hierarchical strategy of testing is suggested. An international consensus on the possible strategy to exclude CF in adults with bronchiectasis is still missing.

The lack of common ground in testing bronchiectasis patients for CF has plenty of clinical and research implications. From a clinical perspective, CF testing seems to be inadequate among bronchiectasis patients. The national British Thoracic Society bronchiectasis audit reported testing for CF in 12% of the population [18], while this percentage was even lower (5.5%) in a similar audit performed in Italy [17].

From a research perspective, several randomized controlled trials (RCTs) in bronchiectasis failed to meet their primary end-points and one of the reason might be because they did not account for the disease heterogeneity [25]. This is especially true looking at the exclusion criteria listed in bronchiectasis RCTs over the past 15 years. Only five out of fifty-one trials excluded CF according to either sweat test or genetic analysis, while the majority of the RCTs reported excluding CF patients only according to past medical history [26–30]. Someone could speculate that some of these studies might have included adult patients with mild or even severe CF due to weakness in their exclusion criteria, thus increasing heterogeneity in study population.

## How to rule out CF in adults with bronchiectasis?

Nowadays, most CF cases are easily detected by newborn screening and diagnoses occur in clinically asymptomatic infants [31]. On the contrary, diagnosis of CF in adults can be challenging because of the heterogeneity of clinical manifestations resulting from highly variable levels of CFTR dysfunction and differential exposure to environmental factors [32]. Recently, guidelines for CF diagnosis have been reformulated by an expert committee designated by the United States CF Foundation [33]. According to this document, CF is established when a subject shows either positive newborn screening results or clinical manifestations consistent with the disease and evidence of CFTR dysfunction. The latter can be either sweat chloride test or CFTR genetic analysis or CFTR electrophysiologic tests. All CF patients should have a sweat test and CFTR gene analysis performed. CF is unlikely in the presence of a negative sweat test, but individuals with sweat chloride < 30 mmol/L, i.e. negative, may still have CF. The absence of two CF-causing CFTR mutations does not exclude the diagnosis of CF. If only one mutation is identified, an extended CFTR genetic analysis may be taken into account. In adult patients without classical CF diagnostic criteria, a CFTR-related disorder (CFTR-RD) might be considered. According to the last recommendations edited in 2011, a CFTR-RD is defined as a clinical entity associated with CFTR dysfunction that does not fulfill the diagnostic criteria for CF [34]. CFTR-RD is usually related with three major clinical phenotypes: CBAVD (congenital bilateral absence of the vas deferens) with CFTR dysfunction, acute recurrent pancreatitis with CFTR dysfunction and disseminated bronchiectasis with CFTR dysfunction.

The most widely used test in clinical practice to diagnose CF/CFTR-RD are listed in Table 2 and reported below in details.

**Table 2:** Comparison among different tests to measure cystic fibrosis transmembrane conductance regulator (CFTR) ion channel activity

	Qualitative	Quantitative	Validated	Clinical	Research
Sweat test		X	X	X	X
CFTR gene analysis	X		X	X	X
NPD		X	X	X	X
ICM		X	X	X	X
Ratiometric sweat secretion optical test		X			X
Monocyte assay		X			X

*CFTR* cystic fibrosis transmembrane conductance regulator; *NPD* nasal potential difference; *ICM* intestinal current measurement

### Sweat test

Measurement of chloride after sweat induction via pilocarpine iontophoresis is the first-line test to diagnose CF [33]. A chloride concentration above 59 mmol/L is used to define a positive sweat test, while values between 30 and 59 mmol/L are deemed inconclusive and call for further evaluations [33].

### *CFTR* gene analysis

More than 2000 different mutations in CFTR gene have been reported (http://www.genet.sickkids.on.ca/cftr/app) and standard mutation panels contain the most common CF-causing mutations (variable coverage depending on panel composition and ethnic origin of the tested people). Further steps like extensive sequencing of CFTR gene and search for large deletions or insertions may be indicated when two CF-causing mutations are not identified by a first level analysis but high clinical suspicion still remains. The classification of CF-causing mutations is still ongoing [33]; in this respect, the CFTR2 website, originated from a project assessing disease liability of CFTR mutations, is a useful but still incomplete tool, including by November 2017 a total of 374 variants (http://www.cftr2.org).

### CFTR Electrophysiologic testing

Nasal potential difference (NPD) and intestinal current measurement (ICM) are used when sweat tests and mutation analysis are inconclusive and the clinical suspicion persists [33, 35]. NPD is an in vivo test of CFTR functionality by measuring nasal transmucosal voltage potential difference, both in basal conditions and after exposure to different chemicals [36]. ICM is an ex vivo test of CFTR function based on the measurement of transepithelial ion transport in rectal biopsies [37]. Both tests are complex to perform and interpret and are available only in highly specialized centers.

### New assays

Ratiometric sweat secretion optical test is intended to assess CFTR function in vivo by comparison of CFTR-dependent and CFTR-independent sweat secretion [38]. Present versions of the test have been able to discriminate among controls, heterozygotes and CF groups showing a continuum in CFTR impairment [39, 40] CFTR is detectable and is functional in human monocytes. The analysis of cell membrane potential changes after the administration of CFTR agonists is lower in CFTR heterozygous carriers and absent in monocytes isolated from CF patients [41]. Correlation with CFTR activity in epithelia is still unclear and further studies are necessary to understand the full potential of this approach.

Physicians taking care of bronchiectasis patients should be aware that all the above tests might have some limitations in the adult population [42, 43]. Sweat test may be not conclusive and intermediate results may be due to residual CFTR functionality as well as environmental factors (e.g. climate, diet) or test variability [42]. In previous reports sweat chloride concentrations appeared to gradually increase during life [43, 44]. In 2013 Traeger and colleagues described 13,782 sweat test performed over 20 years in a single center and reported a peak during middle age, followed by a decrease among elderly subjects [45]. Furthermore, a cut-off of 30 mmol/L for the definition of ‘CF unlikely’ is still debated because it just lies above the median values for sweat chloride in this population, possibly resulting in a greater number of subjects with ambiguous positive results [45]. Finally, over the last decades sweat test has been largely used among infants, children and younger adults, but a validation in elderly population is still lacking.

Diagnostic accuracy of genetic analysis depends on the testing strategy. CFTR gene panels can be designed to be high in specificity, as they test only for CF-causing mutations, at the cost of a relatively lower sensitivity. Sequencing and testing for deletion/duplication increase sensitivity, but reduce specificity by identifying CFTR variants of unknown clinical meaning.

NPD is affected by a very high intra-test variability and several protocol variations and scores for interpretation are reported among different centers [46, 47]. Furthermore, this method needs an experienced operator (hundreds of tests performed), thus limiting a larger use [48]. ICM has been performed following different protocols and, although discussed, it is not present in current diagnostic algorithm and criteria [33, 37]: validation and reference data have been reported [49] . Standard operating procedures of both NPD and ICM are currently available from European Cystic Fibrosis Society (https://www.ecfs.eu/ecfs_dnwg).

**Figure 1:**
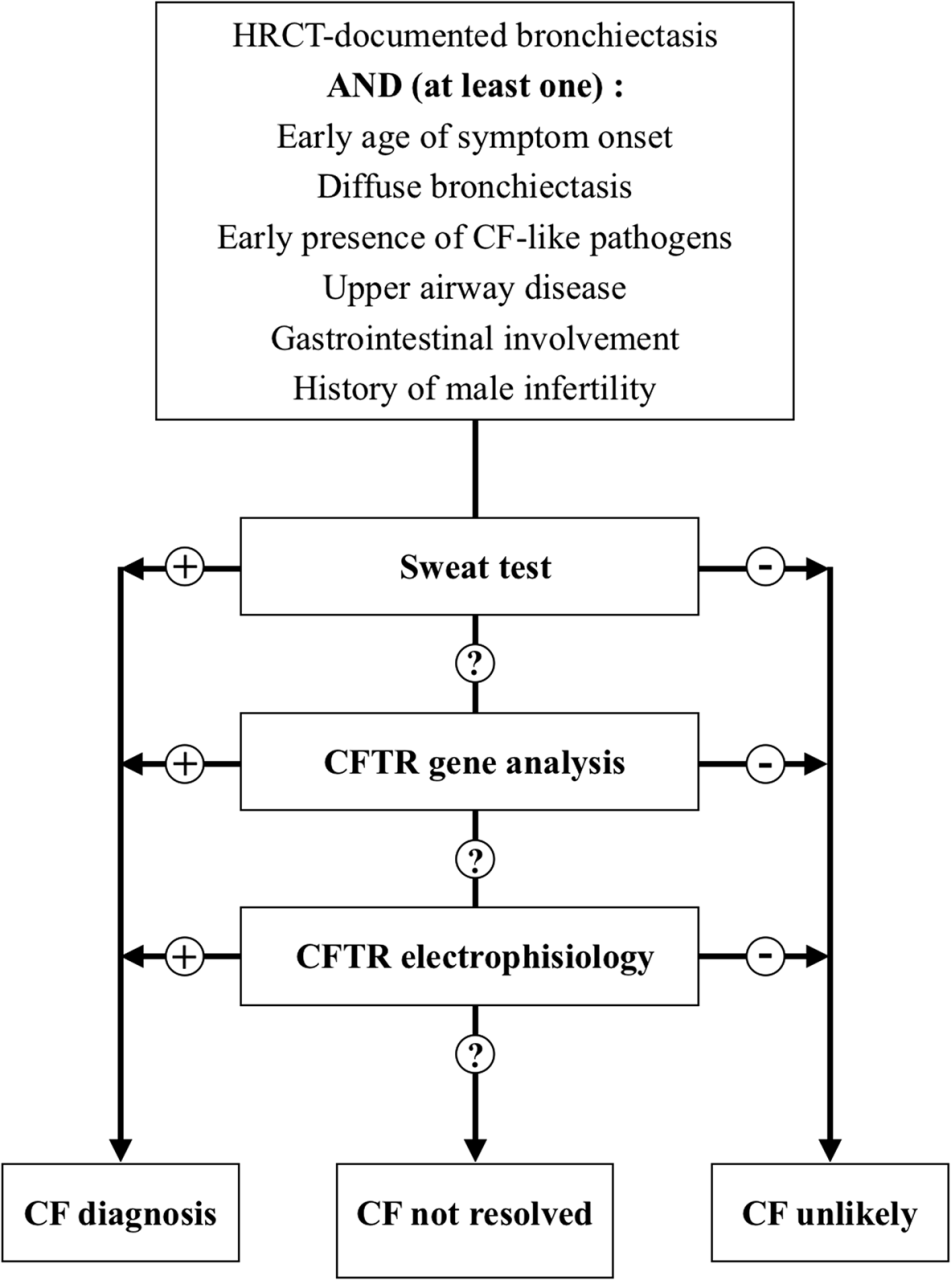
When and how testing patients with bronchiectasis for CF

The new assays as ratiometric sweat secretion optical test have not been validated yet in clinical practice and they are currently used in translational research. Figure 1 reports a flow-chart for when and how to test CF in people with bronchiectasis.

## Implications for future research

Over the last decades, different investigators evaluated the role of CFTR in adults with bronchiectasis. Increased frequency of a single CF-causing mutation in azospermic men was firstly reported by Dumur in 1990 [50]. Since then, a number of papers have been published to investigate the relationship between the presence of a single CFTR mutation and the presence of diffuse bronchiectasis, demonstrating that a CFTR heterozygous condition is more frequent in bronchiectasis patients with normal sweat test in comparison to the general population [51–54]. However, although a diagnosis of CF should be pursued in reason to clinical or social implication, a further speculation on cost/benefit is now needed. The question about how many adults with bronchiectasis need to be tested in order to have a CF diagnosis still remains unanswered. Two scenarios are possible: patients older than 60 years old might not benefit from CF testing in consideration of low catch rate and poor impact on treatment and prognosis; otherwise, CF testing will become cheaper in the future and CFTR screening could be considered.

Also interesting for the discussion is the hypothesis that even a single mutation in CFTR gene may contribute to the development of lung disease. On this side, it has been speculated that bronchiectasis may derive from a complex multifactorial interaction between genetic risk factors and environment. Other genes, so far unidentified, are likely to interact with CFTR activity in the development of the disease; in particular, altered sodium homeostasis due to mutations in ENaC channel is still poorly understood but may play a contributive role [55, 56].

Next step in translational research could be the implementation of a lung genetic panel in bronchiectasis. The strengths of this panel would be a careful selection of genes based on current literature and the inclusion of all those mutations known to be target of new genetic modifiers as well as genes involved in lung inflammation. This approach could lead to new treatments based on better molecular understanding and different therapeutic targets. An individualized approach is necessary to understand the occurrence and contribution of ion channel functional status in bronchiectasis with the aim to evaluate the overall functionality of CFTR and, possibly, other ion channels. As factors not covered by the sequencing of the coding region and the exon/intron junctions could also influence CFTR function, even a sophisticated mutation analysis alone might not identify functionally relevant alterations. Recent developments in the field of stem cell research permit the collection of renewable tissue expressing CFTR and a detailed study of its channel function in organoids derived from minimally invasive rectal biopsies [57]. The use of respiratory cells derived from nasal brush can be envisioned in the near future [58]. These approaches might permit to identify previously undetected alteration in channel homeostasis and unravel novel mechanism/s of disease.

## Conclusions

Ruling out CF is a cornerstone in etiological screening for patients with bronchiectasis and should be implemented in usual work-up for investigating bronchiectasis etiology. CFTR mutations, often with unclear pathogenic effect, have been reported with higher frequency among patients with bronchiectasis. Possibly, a single mutation in CFTR combined with other genetic and environmental factors might be involved in the pathogenesis of bronchiectasis. Further studies on CFTR expression in human lung and traslational research might elucidate the role of CFTR in bronchiectasis.
